# SMAD3 and FTO are involved in miR-5581-3p-mediated inhibition of cell migration and proliferation in bladder cancer

**DOI:** 10.1038/s41420-022-01010-8

**Published:** 2022-04-13

**Authors:** Jiazhu Sun, Xueyou Ma, Yufan Ying, Weiyu Wang, Haixiang Shen, Song Wang, Haiyun Xie, Jiahe Yi, Weitao Zhan, Jiangfeng Li, Ben Liu

**Affiliations:** 1 Department of Urology, The First Affiliated Hospital, Zhejiang University School of Medicine, 310003 Hangzhou, China; 2 Cancer Center, Zhejiang University, 310058 Hangzhou, China

**Keywords:** Bladder cancer, Tumour biomarkers

## Abstract

Previous research evidence suggests that microRNAs (miRNAs) play an indispensable role in onset and progression of bladder cancer (BCa). Here, we explored the functions and mechanisms of miR-5581-3p in BCa. miR-5581-3p, as a tumor suppressor in BCa, was detected at a lower expression level in BCa tissue and cells in contrast with the non-malignant bladder tissue and cells. Over-expression of miR-5581-3p remarkably dampened the migration and proliferation of BCa in vitro and in vivo. SMAD3 and FTO were identified as the direct targets of miR-5581-3p by online databases prediction and mRNA-seq, which were further verified. SMAD3 as a star molecule in modulating EMT progress of BCa had been formulated in former studies. Meanwhile, FTO proved as an N6-methyladenosine (m^6^A) demethylase in decreasing m^6^A modification was confirmed to regulate the migration and proliferation in BCa. In addition, we conducted rescue experiments and confirmed overexpressing miR-5581-3p partially rescued the effects of the overexpressing SMAD3 and FTO in BCa cells. In conclusion, our studies exhibit that miR-5581-3p is a novel tumor inhibitor of BCa.

## Introduction

Bladder cancer (BCa) is a common cancer with newly diagnosed cases ranking the ninth in all kinds of cancers worldwide. In the USA, 64,280 males were diagnosed with BCa in 2021, ranking fourth in the incidence of all cancers and 12,260 males died from BCa, ranking eighth in mortality of all cancers [1]. Non-muscle-invasive BCa (NMIBC) is identified as an early stage of BCa with a better prognosis than muscle-invasive BCa (MIBC). However, the recurrence rate of NMIBC at 5 years is as high as 31% to 78%, and a progression rate of 1% to 45% [2]. Once BCa progresses into metastatic cancer, the 5-year overall survival is only 6% [3]. Hence, it is pivotal to reveal the mechanisms of BCa and create novel treatments to enhance the prognosis of patients.

MicroRNAs (miRNAs) are a cluster of short non-coding RNAs (19–22 nucleotides) that play an indispensable role in modulating the downstream mRNA through specifically docking to its 3′-untranslated region (3′-UTR) [4]. A large quantity of findings has confirmed miRNAs as tumor regulators affects the functions and prognosis of cancers such as BCa. Our research group previously confirmed a series of miRNAs consisting of miR-124-3p, miR-148-3p, miR-193-3p, miR-320c, miR-323-3p, miR-381-3p, miR-22, miR-300, miR-502-5p, miR-608, and miR-665 function as tumor suppressors regulating tumorigenicity, prognosis and progression of BCa [5–15]. Some of them are positioned at DLK1-DIO3 imprinted domain and associated closely with each other [16]. miR-5581-3p is located at chr1p34.3 and was previously documented to function as a tumor promoter in hepatocellular carcinoma and glioma [17, 18]. Nevertheless, the accurate role of miR-5581-3p in BCa remains unclear.

In our study, we discovered that the expression of miR-5581-3p is downregulated in BCa cell lines and tissues. Further studies exhibited that miR-5581-3p is a vital tumor suppressor to dampen the proliferation and EMT progression. Furthermore, we confirmed SMAD3 and FTO were the direct downstream targets of miR-5581-3p. Hence, we propose a novel mechanism induced by miR-5581-3p in BCa that could offer a potential avenue to deal with BCa.

## Results

### miR-5581-3p is a tumor suppressor in BCa

**Fig. 1:**
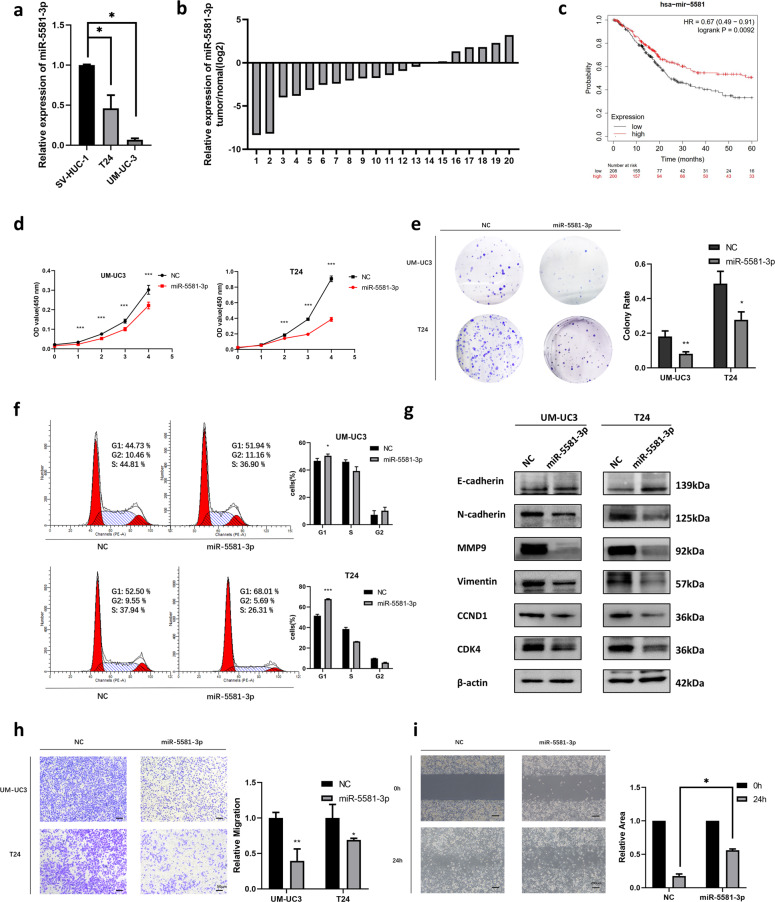
miR-5581-3p is a tumor suppressor in BCa. **a** Relative contents of miR-5581-3p in BCa cells (T24 and UM-UC3) are in contrast with those in non-malignant urothelial cell lines (SV-HUC-1). **b** Comparison of the contents of miR-5581-3p in individual 20 pairs of BCa tissues are presented with the matching neighboring non-malignant tissues. **c** Kaplan–Meier survival data exhibits that upregulation of miR-5581-3p is remarkably related to a high OS rate of BCa. **d** CCK-8 data showed that the relative vitality of cells in the miR-5581-3p (50 nM)-inoculated groups of UM-UC3 and T24 cell lines was lower over time than the NC-inoculated groups. **e** Colony formation assays demonstrated that the rate of colony development was reduced in the miR-5581-3p (50 nM) mimic-inoculated groups compared to the NC-inoculated groups. **f** Cell cycle assays demonstrated over-expression of miR-5581-3p (50 nM) elevated the fraction of cells arrested in G1 phase in UM-UC3 and T24 cell lines. **g** Western blot data verified dampening of migration and proliferation-linked proteins via assessment of over-expression of miR-5581-3p (50 nM) in UM-UC3 and T24 cell lines. **h** Transwell assays revealed miR-5581-3p (50 nM) reduced the migration rate of UM-UC3 and T24 cell lines. **i** Wound-healing assays showed miR-5581-3p (50 nM) dampened the mobility of UM-UC3 cell line. Error bars designate SD acquired from three independent experiments; *P < 0.05, **P < 0.01, ***P < 0.001.

To investigate the miR-5581-3p content in BCa, quantitative reverse transcription PCR (RT-qPCR) assays were performed in UM-UC3 and T24 cell lines. The content of miR-5581-3p was remarkably lower in contrast with the SV-HUC-1 cells (Fig. 1(a)). The data demonstrated a substantial reduction in miR-5581-3p content in BCa tissue specimens compared to non-malignant tissues in 20 BCa pairs of clinical tissue specimens and surrounding non-malignant tissue specimens (Fig. 1(b)). Clinical details of the patients are reported in Table S1. However, no statistically significant relationship was detected between miR-5581-3p expression and the TNM stage of BCa in these samples (Fig. S1). Besides, Kaplan–Meier survival data exhibited high level expression of miR-5581-3p was strongly related to high OS (overall survival) of individuals with BCa (Fig. 1(c)). Given the above findings, we speculated that miR-5581-3p might have a role as a tumor suppressor in BCa. To reveal the dampening effect on proliferation of miR-5581-3p in BCa cell lines, we transfected the cells with miR-5581-3p mimics (50 nM). Cell viability was detected by CCK-8 assays every 24 h from Day 0 to Day 4, and the data exhibited that dampening of cell viability became more apparent over time (Fig. 1(d)). Consistently, colony formation assays illustrated that miR-5581-3p could remarkably decrease the colony formation rate in BCa cell lines (Fig. 1(e)). To investigate the mechanisms of cell growth dampening, we conducted flow cytometry assays. The G1 phase arrest caused by miR-5581-3p was observed in BCa cell lines (Fig. 1(f)). Simultaneously, as crucial modulation factors of the G1/S transition, the expression of CDK4 and CCND1 were also inhibited (Fig. 1(g)). As a result, the over-expression of miR-5581-3p remarkably dampened cell growth in BCa cells through G1 phase arrest. Additionally, transwell assays illustrated that a remarkable dampening of cell mobility was documented in the miR-5581-3p-transfected group (Fig. 1(h)). Wound-healing assays were also conducted to prove overexpressing miR-5581-3p reduced the wound-healing ability of BCa cells (Fig. 1(i)). Simultaneously, miR-5581-3p over-expression inhibited the expression of MMP9 and the EMT process by regulating the expression level of associated proteins (Fig. 1(g)). These findings indicated that the over-expression of miR-5581-3p remarkably dampened the proliferation and the migration ability of BCa cell lines.

### SMAD3 and FTO are determined as the direct targets of miR-5581-3p

**Fig. 2:**
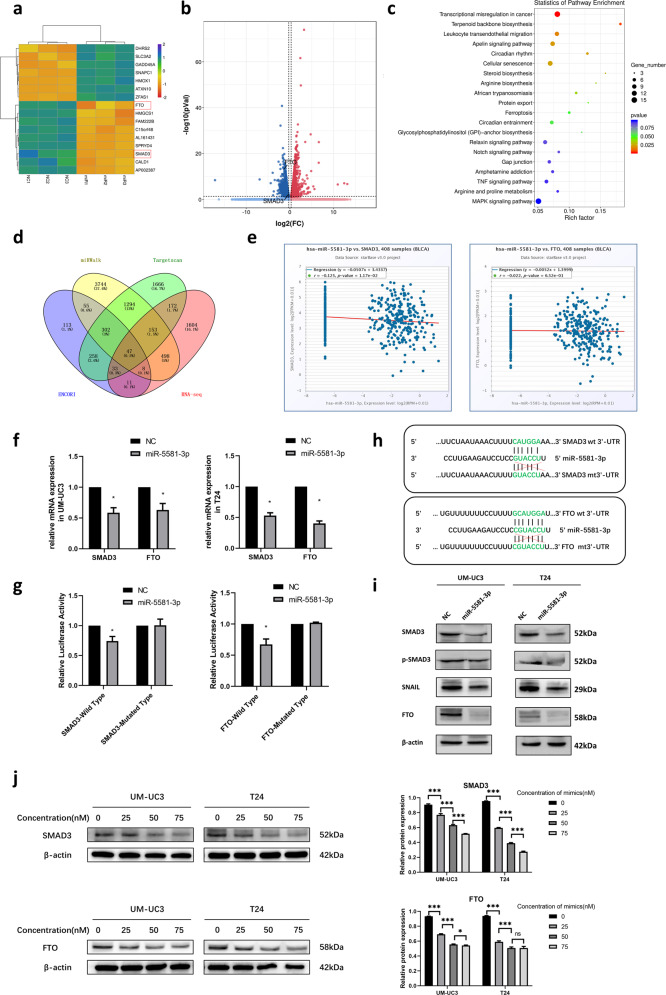
SMAD3 and FTO are determined as the direct targets of miR-5581-3p. **a** Heatmap assessed from mRNA-seq data listed a part of potential downstream genes of miR-5581-3p. **b** Volcano plot illustrated enrichment of remarkably differentially expressed genes in miR-5581-3p overexpressed vs. control UM-UC3 cells. **c** KEGG pathways enrichment from mRNA-seq data. **d** Venn diagram illustrated the intersected genes from mRNA-seq and online databases including miRWalk, TargetScan, and ENCORI. **e** The relative content of SMAD3 and FTO in BCa were associated with miR-5581-3p in ENCORI database. **f** A significant downregulation of SMAD3 and FTO were detected via qRT-PCR assay in UM-UC3 and T24 cell lines post over-expression of miR-5581-3p. **g** Dual-luciferase enzyme reporter assay illustrated that miR-5581-3p remarkably reduced the luciferase enzyme activity of vectors harboring the 3′-UTRs of SMAD3 and FTO. **h** Schematic diagram illustrating the miR-5581-3p-targeting sites of SMAD3 and FTO with seed matching. **i** Western blot data demonstrating decreased protein concentration of SMAD3, FTO, and other proteins in cells transfected with miR-5581-3p mimics. **j** Western blot data demonstrated the protein levels of SMAD3 and FTO changed in BCa cells transfected of miR-5581-3p mimics at the concentration of 0, 25, 50, and 75 nM. Error bars designate SD acquired from three independent experiments; *P < 0.05, **P < 0.01, ***P < *0.001*.

Typically, miRNAs can particularly dock to the 3ʹ-UTR of target mRNAs and suppress the expression level of target genes. To determine direct targets of miR-5581-3p, transcriptome sequencing (mRNA-seq) was performed in miR-5581-3p overexpressed UM-UC3 cells and NC UM-UC3 cells. The result of mRNA-seq was shown in supplementary Table 2. Differentially expressed genes were selected and a part of the genes were shown as a heatmap and a volcano plot (Fig. 2(a, b)). These genes were mainly enriched in transcriptional misregulation in cancer, terpenoid backbone biosynthesis, leukocyte transendothelial migration, apelin signaling pathway and et al. (Fig. 2(c)). The downregulated genes in the miR-5581-3p group were picked out and regarded as the prospective targets of miR-5581-3p. Meanwhile, bioinformatics prediction websites (TargetScan, ENCORI and miRWalk) were utilized for predicting the target genes. Forty-seven candidate genes including SMAD3 and FTO were identified combining the results of mRNA-seq and database prediction (Fig. 2(d)). SMAD3 was negatively correlated with miR-5581-3p in the ENCORI database (*P* < 0.05) (Fig. 2(e)). Although FTO was not remarkably negatively correlated with miR-5581-3p in the ENCORI database (*P* > 0.05), we were interested in it as it acted as an essential m^6^A eraser regulating mRNA methylation [19] (Fig. 2(e)). To confirm SMAD3 and FTO were the target genes of miR-5581-3p, qRT-PCR assays were performed. It was showed that mRNAs of SMAD3 and FTO were downregulated after transfection with miR-5581-3p mimics (Fig. 2(f)). Hence, we further verified whether SMAD3 and FTO were the direct targets of miR-5581-3p by dual-luciferase enzyme reporter assays. The 3ʹ-UTR of SMAD3 and FTO were cloned into pmirGLO Dual-Luciferase Enzyme miRNA Target Expression Vectors respectively. A significant reduction of the relative luciferase enzyme activity of SMAD3 and FTO were detected when miR-5581-3p was overexpressed in UM-UC3 cells. The luciferase enzyme activity of the mutated group did not drop as much as that of the wild group (Fig. 2(g)). The targeted and modified sequences were depicted schematically (Fig. 2(h)). Western blot assays exhibited the protein level of SMAD3 and FTO were remarkably reduced after miR-5581-3p mimic treatment. The protein level of phosphorylation of SMAD3 (p-SMAD3) and SNAIL were also downregulated by miR-5581-3p (Fig. 2(i)). In addition, we used varying concentrations of mimics ranging from 0 to 75 nM in BCa cells. Western blot studies revealed that at 50 nM, the protein levels of SMAD3 and FTO were ~40%–50% decreased. The protein levels of FTO did not change significantly when the concentration was increased to 75 nM in BCa cells compared to 50 nM. Although the protein levels of SMAD3 still decreased when the concentration was increased to 75 nM, the concentration of 50 nM was high enough to suppress the SMAD3 protein expression efficiently. As a result, 50 nM was deemed the most acceptable concentration of mimics that efficiently decreased target gene expression (Fig. 2(j)). As a result, these data showed that SMAD3 and FTO were the direct downstream targets of miR-5581-3p.

### Silencing FTO inhibits the cell migration and proliferation of BCa cell lines

**Fig. 3:**
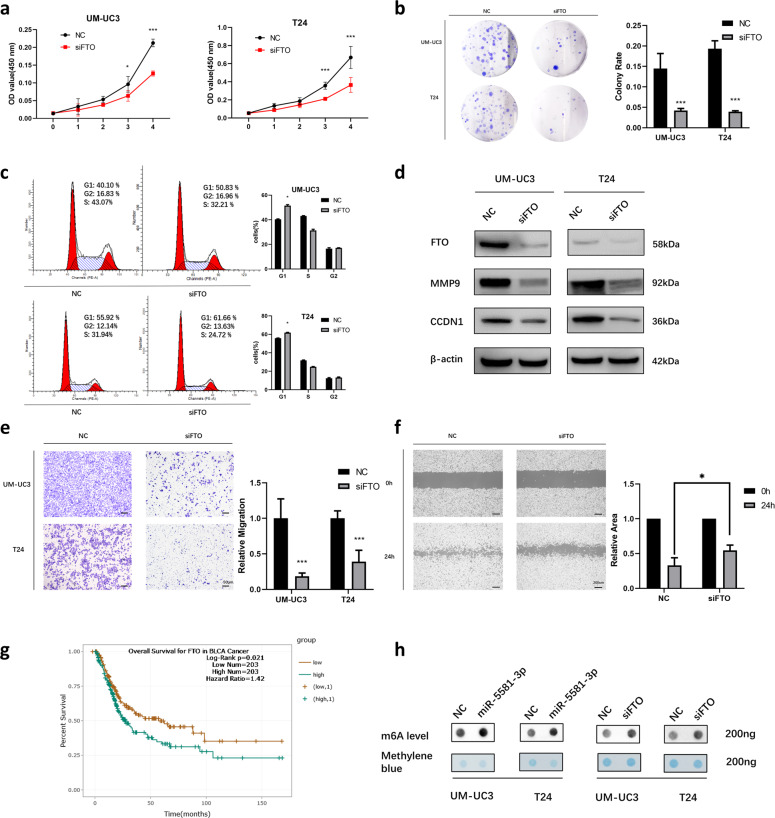
FTO silencing reduces cell migration and proliferation in BCa cell lines. **a** CCK-8 assays demonstrated that the relative vitality of cells from siFTO (50 nM)-inoculated groups of UM-UC3 and T24 cell lines was lower over time than that of NC-inoculated groups. **b** Colony formation assays illustrated that the colony formation rate was lower for the siFTO (50 nM)-inoculated groups in contrast with that of the NC-inoculated groups. **c** Cell cycle assays demonstrated inhibition of FTO (50 nM) elevated the fraction of cells arrested in G1 phase in UM-UC3 and T24 cell lines. **d** Western blot data confirmed dampening of migration and proliferation-linked proteins were detected by the inhibition of FTO (50 nM) in UM-UC3, as well as T24 cell lines. **e** Transwell assays revealed siFTO (50 nM) reduced the migration rate of UM-UC3 and T24 cell lines. **f** Wound-healing assays showed siFTO (50 nM) inhibited the mobility of UM-UC3 cell line. **g** Kaplan–Meier survival data exhibits that upregulation of FTO is remarkably related to a low OS rate of BCa. **h** Dot-blot data indicated that the m^6^A content of UM-UC3 and T24 cell lines changed with the expression of miR-5581-3p and FTO. Error bars designate SD acquired from three independent experiments; *P < 0.05, **P < 0.01, ***P < 0.001.

The CCK-8 assay revealed the knockdown of FTO by siFTO suppressed the cell viability of BCa cell lines (Fig. 3(a)). Consistently, colony formation assays showed that silencing FTO remarkably decreased the colony formation rate in BCa cell lines (Fig. 3(b)). G1 phase arrest induced by siFTO in BCa cell lines was remarkably discovered (Fig. 3(c)). Simultaneously, the expression of CCND1 was also inhibited (Fig. 3(d)). Consequently, the silencing of FTO remarkably inhibited cell growth in BCa cells through G1 phase arrest. In addition, transwell assays and wound-healing assays indicated that a remarkable dampening of cell mobility was found in the siFTO transfected group (Fig. 3(e, f)). Simultaneously, silencing FTO inhibited the expression level of MMP9 (Fig. 3(d)). In addition, Kaplan–Meier survival data exhibited that high level expression of FTO was strongly related to a low OS rate of BCa (Fig. 3(g)). These findings indicated that the silencing of FTO remarkably dampened the proliferation and migration capacity of BCa cell lines.

### Overexpressing miR-5581-3p or silencing FTO changes the total RNA m^6^A level in BCa cell lines

As an m^6^A demethylase, FTO plays an important role in mRNA demethylation. We hypothesized that miR-5581- 3p changes the m^6^A levels by targeting FTO directly and regulates the progression of BCa. To confirm the hypothesis, dot-blot assays were performed to identify changes in m^6^A levels in BCa cells transfected with miR-5581- 3p mimics or siFTO. The data showed that the total RNA m^6^A levels were remarkably reduced in the miR-5581-3p or siFTO groups (Fig. 3(h)).

### Over-expression of SMAD3 and FTO partially rescues the miR-5581-3p-inhibited mobility of BCa cells

**Fig. 4:**
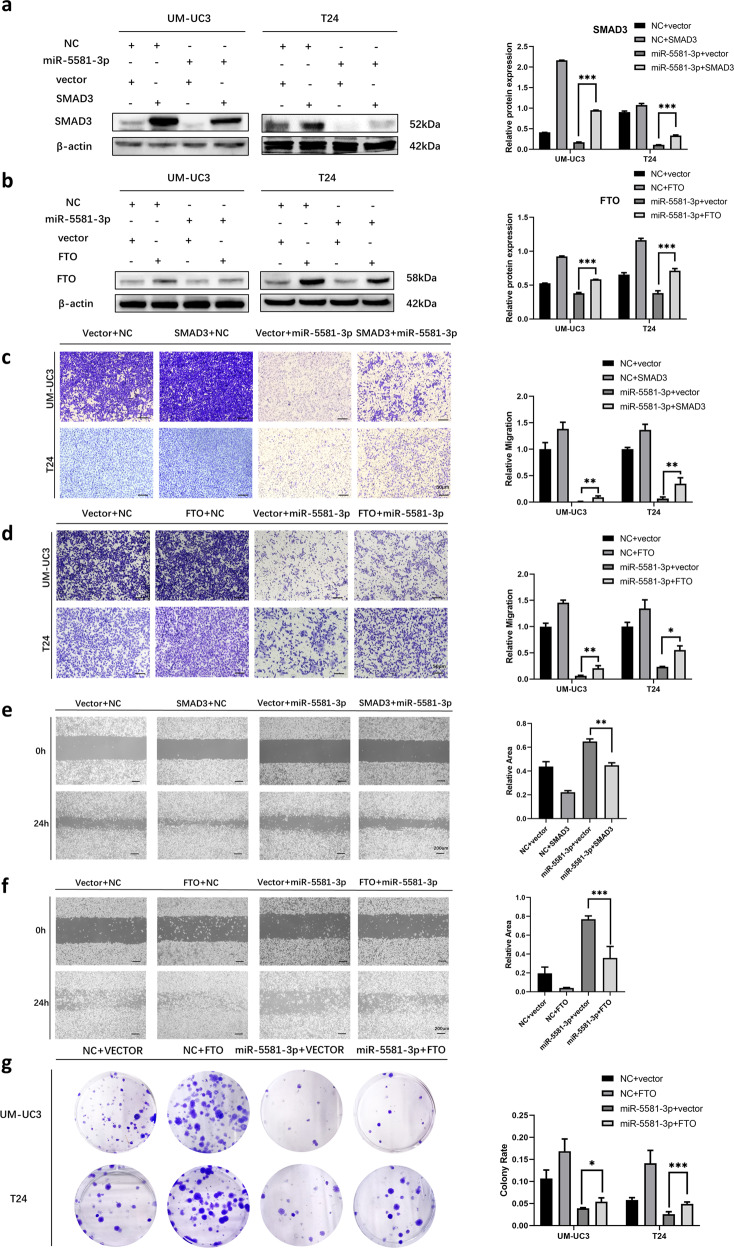
Overexpression of SMAD3 and FTO partially restores BCa cells motility and proliferation that was reduced by miR-5581-3p. **a** Western blot assays showed co-transfection of miR-5581-3p and SMAD3 rescued the protein levels of SMAD3 in UM-UC3 and T24 cell lines. **b** Western blot data showed co- transfection of miR-5581-3p and FTO rescued the protein levels of FTO in UM-UC3 and T24 cell lines. **c** Transwell assays consistently showed miR-5581-3p-induced cell migration inhibition was partly rescued by SMAD3 over-expression. **d** Transwell assays consistently showed miR-5581-3p-induced cell migration inhibition was partially rescued by FTO over-expression. **e** The wound-healing assays exhibited that the miR-5581-3p-triggered cell mobility dampening was partly rescued via SMAD3 over-expression. **f** The wound-healing assays illustrated that the miR-5581-3p-triggered cell mobility dampening was partly rescued via FTO over-expression. **g** Colony formation assays illustrated that the co-transfection of miR-5581-3p and FTO rescued the growth dampening triggered by miR-5581-3p over-expression. Error bars designate SD acquired from three independent experiments; *P < 0.05, **P < 0.01, ***P < 0.001.

SMAD3 as a pivotal gene in modulating the EMT progression of BCa has been reported in our previous study [5, 15]. These studies confirmed knockdown of SMAD3 dampened the migration of BCa cell lines and affected the EMT-associated protein expression level. To further investigate the direct cross-talk of SMAD3 with miR-5581-3p, we carried out rescue experiments with a SMAD3 overexpressed plasmid. Meanwhile, the rescue experiments between FTO and miR-5581-3p were also performed to verify their direct interaction. We performed Western blot assays to examine the SMAD3 and FTO expression levels, which showed the change as expected (Fig. 4(a, b)). The transwell and wound-healing assays exhibited that over-expression of SMAD3 or FTO enhanced migration ability of BCa cells lines. Moreover, co-transfection of the SMAD3 or FTO plasmid and miR-5581-3p significantly reversed the suppressed migration potential induced by miR-5581-3p in BCa cell lines (Fig. 4(c–f)). Moreover, the colony formation assays indicated co-transfection of the FTO plasmid with miR-5581-3p remarkably reverted the suppressed proliferation ability triggered by miR-5581-3p in BCa cell lines (Fig. 4(g)). These results indicated that SMAD3 and FTO, as direct targets of miR-5581-3p, played a crucial role in BCa migration and proliferation.

### miR-5581-3p played a tumor-suppressing role in vivo

**Fig. 5:**
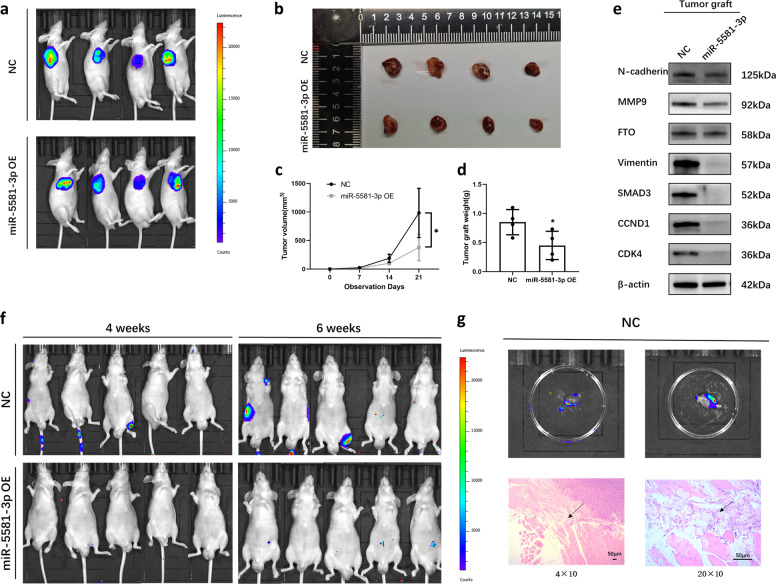
miR-5581-3p plays a tumor-suppressing role in vivo. **a** The luciferase enzyme activity of the miR-5581-3p group compared to the NC group by fluorescent microscopic image analysis. **b**–**d** Subcutaneous xenograft model demonstrated the tumor growth and weight of xenograft tumors in mice. **e** Western blot assay showed downstream proteins of miR-5581-3p were downregulated in the miR-5581-3p-transfected group. **f** A metastasis model of the BALB/c nude mice by tail vein injection indicated the metastatic capacity of tumors in fluorescence activity and the quantity of metastasis sites. **g** The metastatic organs were imaged to re-verify the metastasis in vivo after the mice were sacrificed. H&E staining of the metastatic organs identified the metastasis position. Error bars designate the SD acquired from at least three independent experiments; *P < 0.05, **P < 0.01, ***P < 0.001.

We established mouse models inoculated with the UM-UC3 cell line to assess the function of miR-5581-3p in vivo. In contrast to the NC group, the miR-5581-3p group had slower tumor growth rate and lower tumor weight (Fig. 5(a–d)). To identify the miR-5581-3p targets, we isolated protein of xenografts and carried out a Western blot assay. The data exhibited that the content of SMAD3, FTO and related downstream proteins were lower in the miR-5581-3p group (Fig. 5(e)). To verify the tumor metastasis-suppressing effect of miR-5581-3p in vivo, we developed a mouse model of experimental metastases by injecting stably transfected UM-UC3 cells into the tail vein. After observing for 6 weeks, the fluorescence activity and the count of metastatic areas diminished in the miR-5581-3p group in contrast with the NC group, indicating dampening of metastasis (Fig. 5(f)). In the H&E staining data, the organs acquired from the NC group were confirmed as metastases (Fig. 5(g)).

## Discussion

**Fig. 6:**
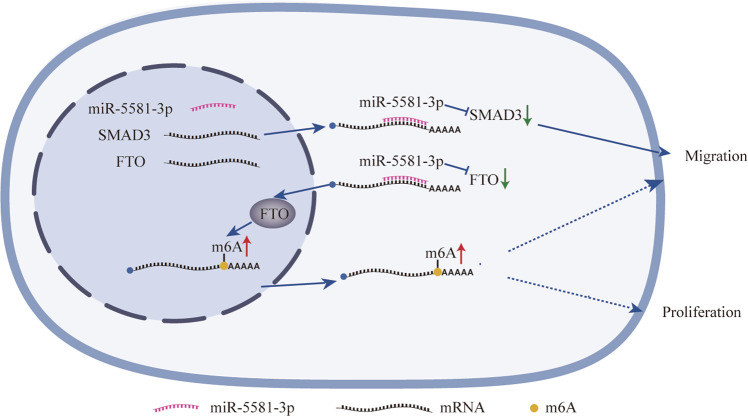
The schematic diagram shows the findings of this study. miR-5581-3p inhibits the expression of SMAD3 and FTO via binding to the 3′-UTR of their mRNAs. Inhibition of SMAD3 suppresses the migration of BCa cells, and inhibition of FTO suppresses the migration and proliferation of BCa cells via m^6^A modification.

Recent research evidence revealed that miRNAs appeared to be important modulators of BCa. Their expression is frequently altered in BCa, and many miRNAs are functionally implicated in the pathogenesis of BCa [20]. The functions of miR-5581-3p were previously reported in HCC, glioma and hemophilia A [17, 18, 21]. Yin et al. and Yan et al. found miR-5581-3p played oncogenic roles as the downstream target of distinct lncRNAs to promote the HCC and glioma progression [17, 18]. Nonetheless, to the best of our knowledge, no research works have been conducted on the distinct role and the mechanism of miR-5581-3p in BCa. Herein, we found that the content of miR-5581-3p in BCa cell lines and tissues was notably lower as a result of epigenetic mechanisms. However, no significant correlations between miR-5581-3p and TNM stages. Maybe it was because there were other confounding factors that influenced the effect of miR-5581-3p on TNM stages. It could also be due to insufficient sample size. We confirmed that the over-expression of miR-5581-3p remarkably suppressed proliferation and migration in BCa cells via modulating EMT and cell cycle pathways. Besides, we examined the subsequent mechanisms of miR-5581-3p in BCa and confirmed SMAD3 and FTO were direct targets of miR-5581-3p. Moreover, we proved m^6^A modification could be engaged in the mechanisms of miR-5581-3p in BCa by targeting FTO (Fig. 6).

An EMT constitutes a biologic process allowing a polarized epithelial cell to go through numerous biochemical changes that make it assume a mesenchymal cell phenotype increased resistance to apoptosis and elevated generation of ECM constituents [22]. It was closely associated with the migration ability of cancer cells [23]. E-cadherin, a key biomarker of EMT plays a role as a tumor suppressor, while N-cadherin and Vimentin act as tumor promotors [24]. Our previous studies had proved that miRNAs could impact the migration ability of BCa cell lines by EMT progression [5–7, 9, 25]. Herein, we confirmed over-expression of miR-5581-3p elevated the content of E-cadherin and suppressed the expression of N-cadherin and Vimentin, just as we were expected.

Several molecules are regarded as EMT-associated inducers. However, only a few cascades are involved in the EMT progression. Among the EMT triggers, TGF-β has been better characterized relative to other triggers, and frequently works as a paradigm for assessment of this process. TGF-β induces SMAD coupled with non-SMAD signaling cascades in EMT. SMADs have the remarkable capacity to cooperate with other transcription-modulated signaling cascades in the control of gene re-programming during EMT [26]. These cascades target SMADs for phosphorylation or other modifications, and thus define their function. Activated TGF-β signaling cascades phosphorylate SMADs and phosphorylated SMADs can regulate a series of downstream biological changes [27]. As stated, SMAD3 serves as a key member of the SMAD family. Our recent investigation in BCa revealed that silencing SMAD3 inhibited the motility of T24 and UM-UC3 cell lines by inhibiting EMT progression.

Meanwhile, phosphorylated SMAD3 (p-SMAD3) expression was also reduced, indicating that activated SMAD3 was lowered [5, 15]. Our findings demonstrated that miR-5581-3p significantly lowered the expression of SMAD3 and p-SMAD3, indicating that miR-5581-3p inhibits the activated form of SMAD3. The dual-luciferase enzyme reporter assays and rescue tests also demonstrated that miR-5581-3p was a direct target of SMAD3. To summarize, we discovered that miR-5581-3p inhibits the migratory potential of BCa cell lines via EMT progression and that SMAD3 is a direct target of miR-5581-3p.

FTO, considered as an N6-methyladenosine (m^6^A) demethylase, is discovered to play an oncogenic or tumor-suppressive role in multiple cancers [28]. One study showed FTO inhibited the progression of BCa [29], while other studies, consistent with our results, confirmed FTO played a role as an oncogene promoting proliferation and migration of BCa cell lines [30–32]. Zhou et al. confirmed FTO regulated the cell cycle of BCa cell lines by indirectly targeting CDK6 via m^6^A modification, while they did not figure out the mechanism of migration promotion ability induced by FTO [32]. MMP9 is an essential enzyme causing degradation of the basement membrane along with the extracellular matrix, regulating migration and metastasis of cancers [33]. In our study, miR-5581-3p remarkably suppressed the expression of FTO. The dual-luciferase reporter assays and rescue experiments also proved FTO was the direct target of miR-5581-3p. Besides, we confirmed MMP9 was the downstream protein of FTO regulating the migration of BCa. Meanwhile, we discovered FTO influenced cell cycle by regulating CCND1, which was also a member of cyclin-dependent kinase family as CDK6 [34]. Besides, we also found overexpressing miR-5581-3p downregulated the expression of MMP9 and CCND1, consistent with the results of silencing FTO. As an eraser of m^6^A modification, FTO has been reported to reduce the m^6^A level in BCa [30–32]. In this study, we confirmed silencing FTO or overexpressing miR-5581-3p would increase the m^6^A level in BCa, suggesting miR-5581-3p regulating the m^6^A modification via FTO. Moreover, a high level of miR-5581-3p or a low level of FTO indicated a high overall survival rate, suggesting FTO might be the key factor influencing the prognosis of miR-5581-3p.

In summary, we investigated the function of miR-5581-3p in BCa and revealed it inhibited the migration and proliferation of BCa cell lines. SMAD3 plays a role as the direct target of miR-5581-3p regulating the migration and FTO was also the direct target of miR-5581-3p regulating the migration and proliferation of BCa via m^6^A modification. In the future, it is possible to recruit miR-5581-3p as an innovative BCa target for precision therapy based on the function and mechanism of miR-5581-3p confirmed in our study.

## Materials and methods

### Cell lines and cell culture

UM-UC3, T24 human BCa cells, the non-malignant bladder cell line SV-HUC-1, and the human embryonic kidney cell line 293T were purchased from the Cell Bank of the Chinese Academy of Science (Shanghai, China). UM-UC3 cell lines were inoculated in MEM medium (Corning); T24 cells were inoculated in RPMI 1640 medium (Corning); SV-HUC-1 cell line was inoculated in F-12K medium (Gibco, Thermo Fisher Scientific); and 293T cell line was maintained in Dulbecco’s modified Eagle medium (Corning). The media were supplemented with 10% heat-inactivated fetal bovine serum (FBS). All cell lines were cultivated at 37 °C and 5% CO_2_ conditions.

### Human clinical samples

We collected twenty paired BCa tissues coupled with neighboring non-malignant bladder mucosal tissues from patients who were treated with BCa radical cystectomy or Transurethral Resection of Bladder Tumor (TURBT) at the First Affiliated Hospital of Zhejiang University from January 2011 to December 2021, after obtaining informed consent from the subjects and Ethics Committee approval. We snap-froze all the tissue specimens in liquid nitrogen waiting for RNA isolation.

### Reagents and transfection

The RNA duplexes were commercially purchased from RiboBio (Shanghai, China). The corresponding sequences are as followed: The miR-5581-3p mimic: 5′-UUCCAUGCCUCCUAGAAGUUCC-3′; the negative control duplex (NC): 5′-ACUACUGAGUGACAGUAGA-3′; siFTO: 5′-AAAUAGCCGCUGCUUGUGATT-3′. The over-expression plasmids pSMAD3, pFTO and negative control pNull were purchased from GeneChem Company. Polyplus transfection® reagent was adopted in transfecting the RNA duplexes and created plasmids as described by the manufacturer (Proteintech) in the gain-of-function or loss-of-function assays, rescue experiments, and dual-luciferase enzyme reporter assays.

### RNA isolation and RT-qPCR

RNA isolation from BCa cell lines and clinical tissue specimens was done with the RNAiso Plus Reagent (Takara). Generation of cDNA from RNA was done using the One Step PrimeScript miRNA cDNA Synthesis Kit and the PrimeScript RT Reagent Kit (Takara). Comparison of transcript and miRNA contents was assessed via running qPCR on the ABI 7500 fast real-time PCR Platform (Applied Biosystems) with the SYBR Premix Ex Taq (Takara), with U6 small nuclear RNA and GAPDH mRNA serving as the normalization standards of miRNA and mRNA, respectively. The 2^−ΔΔCt^ approach was adopted to explore relative expression. All primers used are listed: miR-5581-3p 5′-TTCCATGCCTCCTAGAAGTTCC-3′; FTO F 5′-ACTTGGCTCCCTTATCTGACC-3′; FTO R 5′-TGTGCAGTGTGAGAAAGGCTT-3′; SMAD3 F 5′-TGGACGCAGGTTCTCCAAAC-3′; SMAD3 R 5′- CCGGCTCGCAGTAGGTAAC-3′; GAPDH F: 5′- AAGGTGAAGGTCGGAGTCA-3′; GAPDH R: 5′-GGAAGATGGTGATGGGATTT-3′; U6 F 5′-TGCGGGTGCTCGCTTCGGCAGC-3′.

### Dual-luciferase reporter assay

We designed and purchased the pmirGLO Dual-luciferase enzyme miRNA Target Expression Vector (Promega) harboring the prospective miR-5581-3p target portion (wild-type) or mutant target region (mutated type) from Sangon. The UM-UC3 cells were inoculated in 96-well plates, and co-transfection was done with 50 nM miR-5581-3p mimics or NC and 100 ng constructed target reporter pmirGLO. The activity of the relative luciferase enzyme was detected with Berthold Detection System via the dual-luciferase enzyme reporter assay platform (Promega) 48 h after transfection.

### Cell-viability assay

Three thousand UM-UC3 or T24 cells were inoculated in 96-well plates, then transfection was done with the RNA duplex at a concentration of 50 nM. Cell-viability assays were measured every 24 h with Cell Counting Kit-8 (Dojindo Laboratories) as described by the manufacturer.

### Colony formation assay

We inoculated 500 cell per well after transfection in 6-well plates and grew them at standardized growth parameters for 10–14 days. Determination of colony counts was done after fixing in absolute methanol accompanied by staining (in 0.1% crystal violet).

### Cell cycle analysis

After digesting and washing transfected cells in PBS, they were fixed (in 70% ethanol) overnight at 4 °C. DNA staining was performed using the cell cycle staining Kit (#CCS012; Multi Sciences). Thereafter, cell cycle assays were detected by the BD LSRII Flow Cytometer Platform with the FACSDiva software (BD Biosciences). Lastly, we analyzed the data via the ModFit LT 3.2 software (Verity Software House).

### Western blot assay

The steps and procedures of Western blot assays were described in detail previously [8]. The primary antibodies utilized consisted of: anti-N-cadherin, anti-MMP9 (Proteintech Group); anti-β-actin, anti-E-cadherin, anti-VIMENTIN, anti-CCND1, anti-CDK4, anti-SMAD3, anti-p-SMAD3, anti-FTO, and anti-SNAIL (Cell Signaling Technology).

### Transwell assay

Migration capacity of BCa cells was evaluated via the transwell assays with transwell compartments (Millipore). Following transfection, ~5.5 × 10^4^ UM-UC3 cells and 4 × 10^4^ T24 cells were dispersed in serum-free medium (200 µL) and delivered to the surface layer of the compartments. The chambers were placed in a 24-well plate and RPMI-1640 or MEM (800 µL) enriched with 10% FBS was introduced to the lower compartment and inoculated for 24 h under 37 °C. After that, we inoculated the compartments with methanol and crystal violet (0.1%). Imaging was done with the phase-contrast microscope (Olympus) with a 20×objective lens.

### Wound-healing assay

After transfection, the cells were cultivated in six-well plates until they were 100% confluent, and a horizontal wound was made with a micropipette tip. After incubation with serum-free medium for 24 h, wound healing was observed with microscopy. Imaging was done with a phase-contrast microscope (Olympus) with a 5× objective.

### RNA m^6^A dot-blot assay

Isolation of total RNAs from BCa cells was done with the RNAiso plus (Takara). We adjusted the levels of RNAs to 50 ng/μL by using RNase-free water. RNA inoculation buffer, mixture of MOPS, formamide and formaldehyde, was used to eliminate secondary structure of RNAs at 65 °C. In all, 200 ng RNA samples were inoculated with cold 20×SSC (Saline-Sodium Citrate) solution (Sigma-Aldrich) and added on wetted N + membrane (GE health) in dot-blot platform. The membrane was UV cross-linked in an Ultraviolet Crosslinker and stained by 0.02% methylene blue (Sigma-Aldrich) to scan the total RNAs. The membrane was imaged by an imager (Bio-rad) after blocking with 5% nonfat milk and inoculating with m^6^A antibody (Synaptic Systems; 1:2000) and secondary antibody.

### Lentiviruses and infection

The lentiviral vectors expressing miR-5581-3p and empty lentiviral vector were purchased from GeneChem. The vector used was pEZX-MR03 with puromycin resistance. The lentivirus infection was manipulated as described by the manufacturer. Stably infected UM-UC3 cells, which can produce luciferase were selected by puromycin.

### RNA sequencing

Total RNA of UM-UC3 was extracted by Triztol from the miR-5581-3p group and negative control group. RNA-seq and RNA-distinct bioinformatics algorithms were done by LC-Bio.

### Animal experiments

Four-week-old male BALB/c nude mice were used for animal experiments. UM-UC3 cells (2 × 10^6^ cells per mouse) stably overexpressing miR-5581-3p and NC were injected into the mice to establish the subcutaneous implantation model. Tumor size was measured by a caliper every week, and tumor volume was calculated by the formula: *V* = (width^2^ × length × 0.52). As for the tumor metastasis model, UM-UC3 cells (1 × 10^6^ cells per mouse) were injected into each mouse via the tail vein. The subcutaneous implantation model used 8 nude mice, whereas the tumor metastasis model used 10 nude mice. Assessment of tumor size and observation of metastasis tumors were done via intraperitoneal inoculation with 15 mg/mL, XenoLight D-luciferin Potassium Salt (100 μL; PerkinElmer) with the IVIS Spectrum animal imaging Platform (PerkinElmer) in every mouse. Eventually, mice were sacrificed for tumors and metastases. Samples were sent for further H&E staining. All animals were manipulated as per the institutional guidelines granted by the First Affiliated Hospital, School of Medicine, Zhejiang University.

### Statistical analysis

The means ± SE was calculated for the data. Differences between groups were evaluated via the Student’s *t*-test or chi-square test. We implemented data analysis in the SPSS 25.0 software (IBM), with a two-tailed *P* < 0.05 signifying statistical significance.

## Supplementary information

Table S1

Figure S1

Table S2

Original data of Western blot

## Data Availability

All data generated or analyzed during this study are included in this published article and its supplementary information files.
