# Supplementary material for: SMAD3 and FTO are involved in miR-5581-3p-mediated inhibition of cell migration and proliferation in bladder cancer
Source: Cell Death Discov. 2022 Apr 13;8:199. doi: 10.1038/s41420-022-01010-8 (PMC9007965; doi:10.1038/s41420-022-01010-8)
Supplement: Supplementary file 1 — Table S1 [file 41420_2022_1010_MOESM1_ESM.docx]

| Supplemental Material 1. Clinical data of the patients. | | | | |
| --- | --- | --- | --- | --- |
| Patient no. | Sex | Age | TNM stage | Histological grade |
| 1 | Male | 62 | T2N0M0 | III |
| 2 | Male | 60 | T1N0M0 | I |
| 3 | Male | 53 | T1N0M0 | III |
| 4 | Male | 86 | T1N0M0 | III |
| 5 | Male | 55 | T1N0M0 | II |
| 6 | Female | 74 | T2N0M0 | III |
| 7 | Male | 56 | T2N0M0 | III |
| 8 | Female | 76 | T3N0M0 | III |
| 9 | Male | 65 | T2N0M0 | II |
| 10  11  12  13  14  15  16  17  18  19  20 | Male  Male  Female  Male  Male  Male  Male  Male  Female  Male  Male | 76  73  61  60  78  67  65  73  76  81  82 | T3N0M0  T2N0M0  T2N0M0  T1N0M0  T2N0M1b  T1N0M0  T1N0M0  T3N0M0  T1N0M0  T1N0M0  T1N0M0 | III  II  III  I  I  III  III  III  I  III  III |
