# Supplementary figures and images for: SMAD3 and FTO are involved in miR-5581-3p-mediated inhibition of cell migration and proliferation in bladder cancer
Source: Cell Death Discov. 2022 Apr 13;8:199. doi: 10.1038/s41420-022-01010-8 (PMC9007965; doi:10.1038/s41420-022-01010-8)

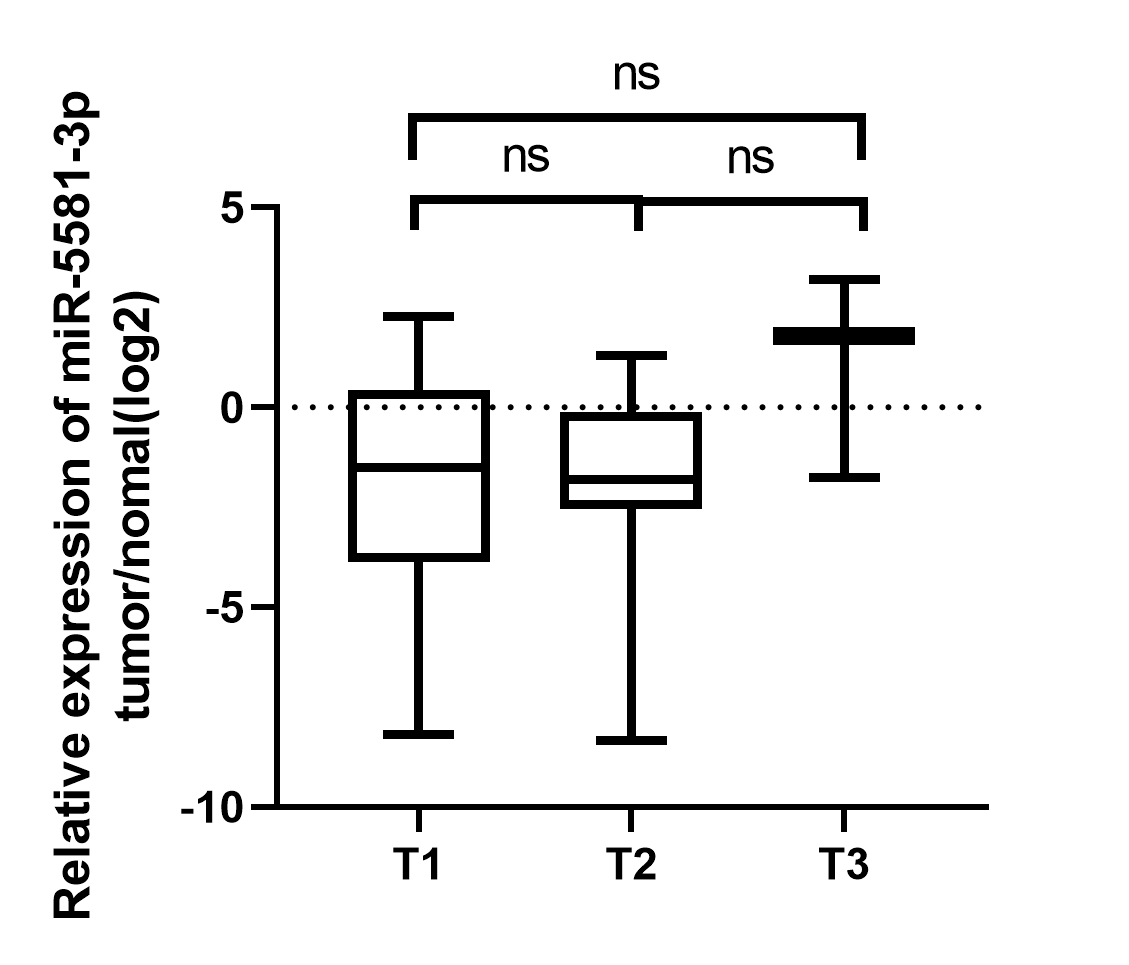

Supplement: Supplementary file 2 — Figure S1 [file 41420_2022_1010_MOESM2_ESM.tif]
