# Supplementary material for: SMAD3 and FTO are involved in miR-5581-3p-mediated inhibition of cell migration and proliferation in bladder cancer
Source: Cell Death Discov. 2022 Apr 13;8:199. doi: 10.1038/s41420-022-01010-8 (PMC9007965; doi:10.1038/s41420-022-01010-8)
Supplement: Supplementary file 4 — Original data of Western blot [file 41420_2022_1010_MOESM4_ESM.docx]

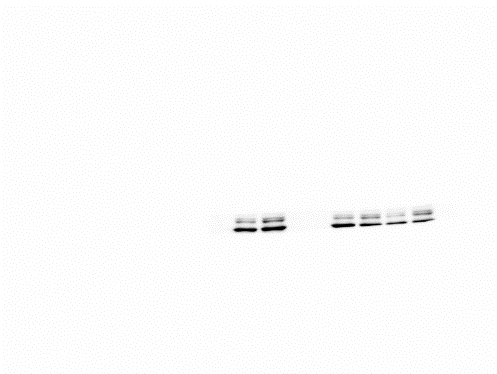


Figure 1 T24 actin


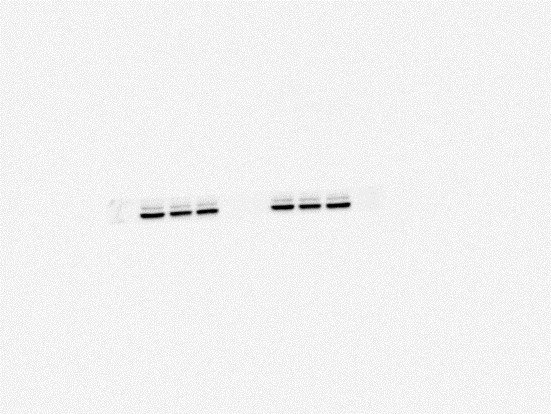


Figure 1 T24 actin2


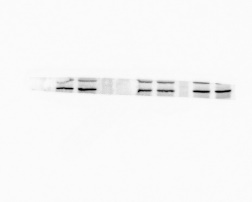


Figure 1 T24 actin3


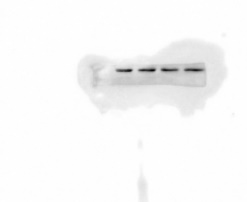


Figure 1 T24 actin4


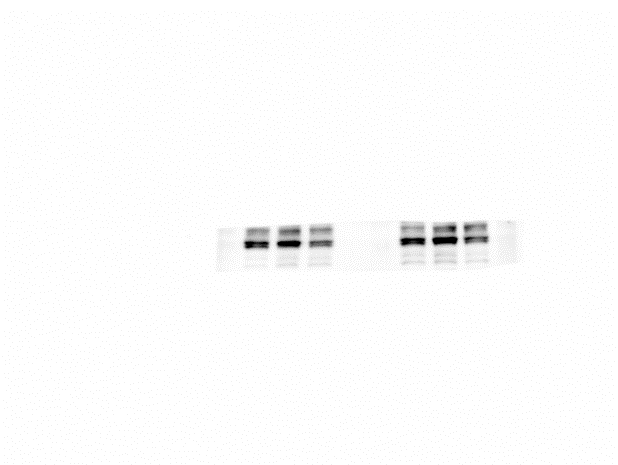


Figure 1 T24 CDK4


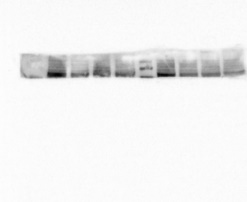


Figure 1 T24 N-Cadherin


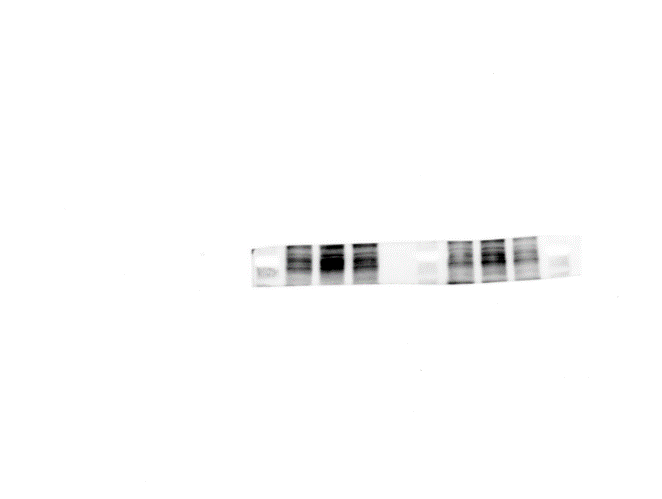


Figure 1 T24 Vimentin


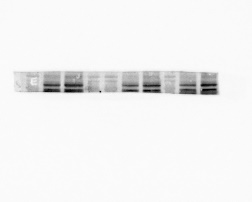


Figure 1 T24 E-Cadherin


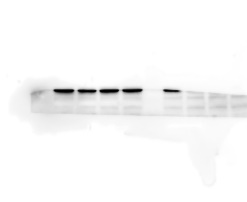


Figure 1 UM-UC3 actin 1


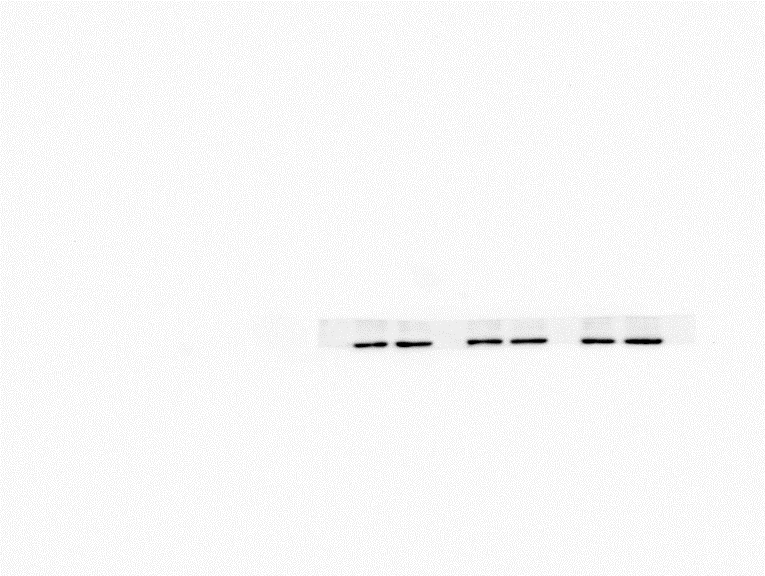


Figure 1 UM-UC3 actin2


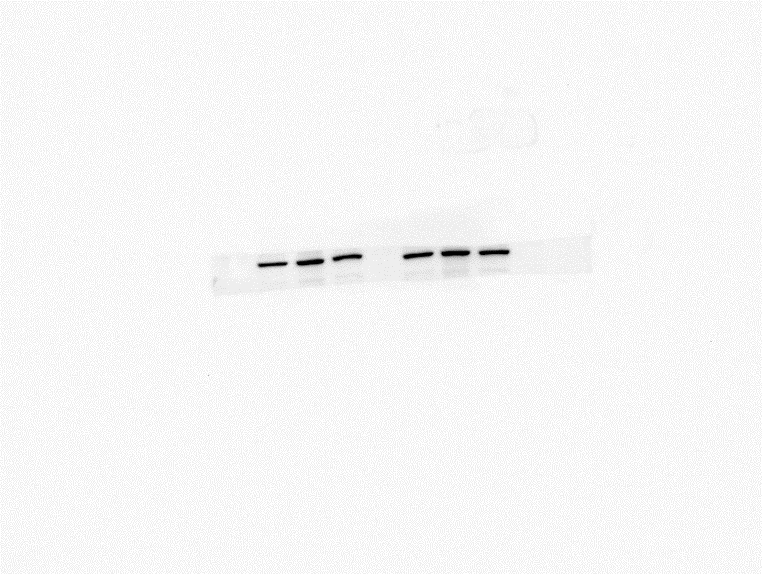


Figure 1 UM-UC3 actin3


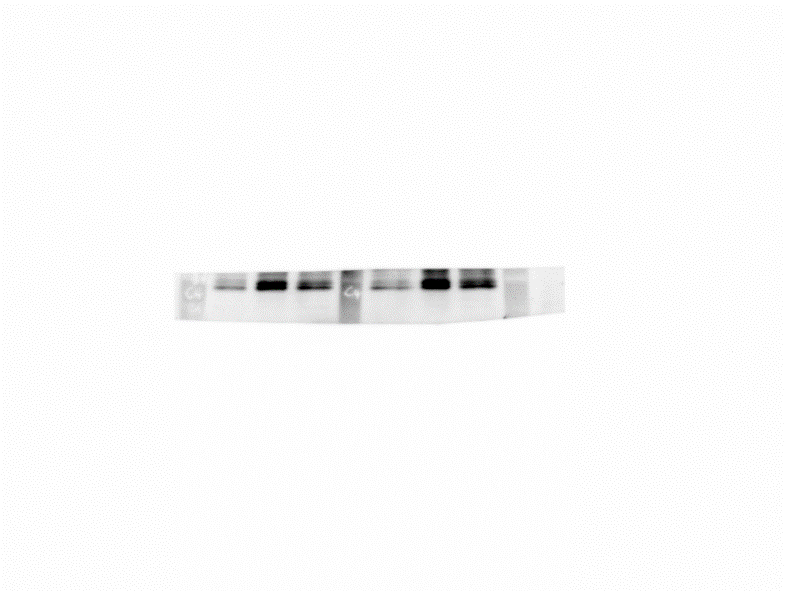


Figure 1 UM-UC3 CDK4


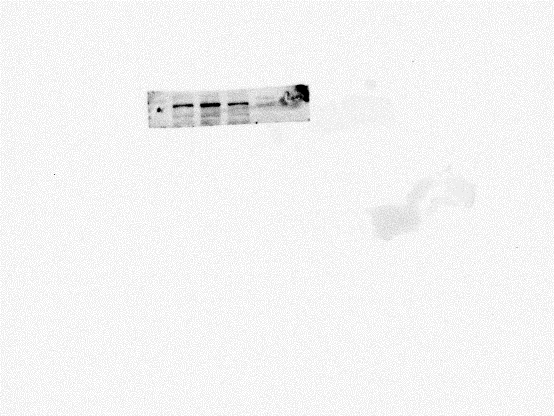


Figure 1 UM-UC3 N-Cadherin


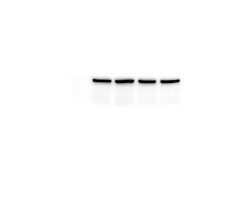


Figure1 UM-UC3 T24 actin


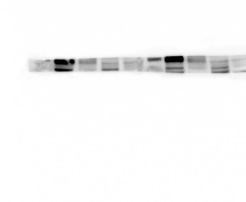


Figure1 UM-UC3 T24 MMP9


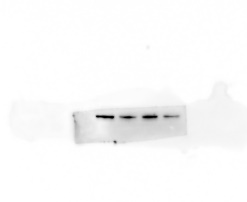


Figure1 UM-UC3 T24 CCND1


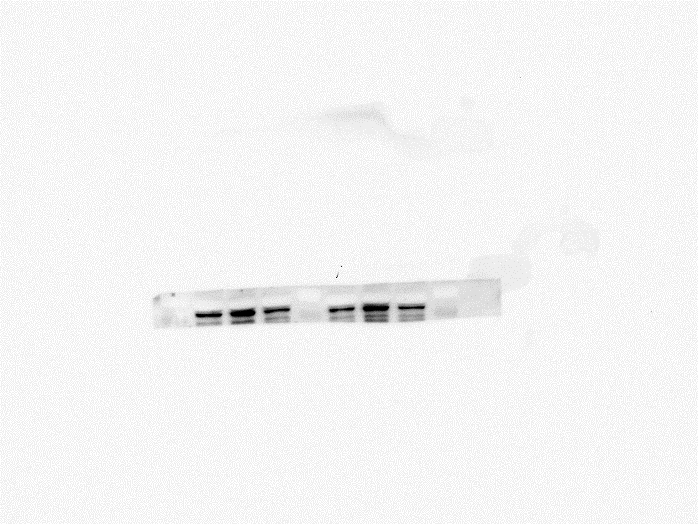


Figure1 UM-UC3 Vimentin


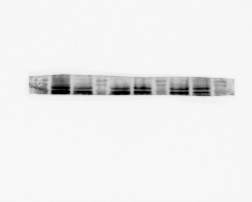


Figure1 UM-UC3 E-Cadherin


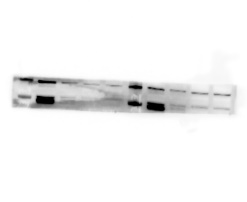


Figure1 UM-UC3 T24 MMP9


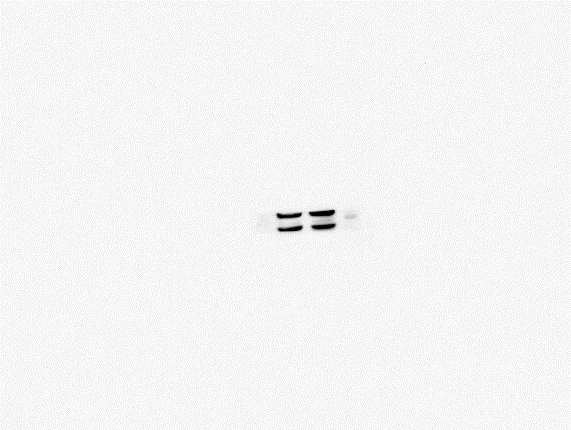


Figure2i T24 actin


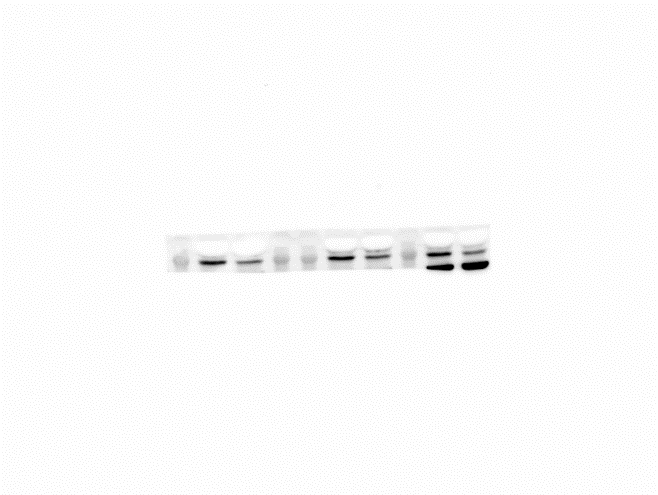


Figure2i T24 SMAD3


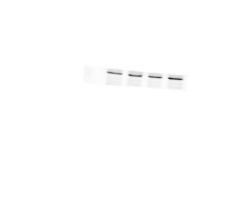


Figure2i UM-UC3 T24 actin


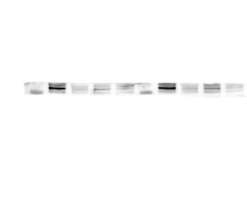


Figure2i UM-UC3 T24 FTO


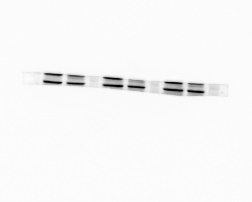


Figure2i UM-UC3 actin


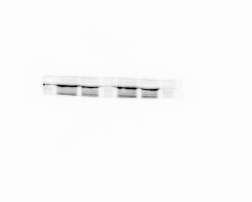


Figure2i UM-UC3 p-SMAD3


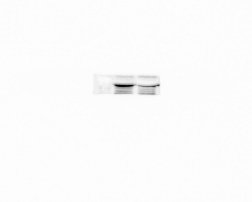


Figure2i UM-UC3 SMAD3


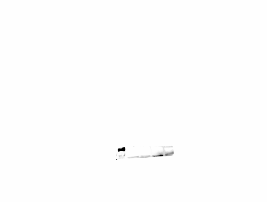


Figure2i T24 p-SMAD3


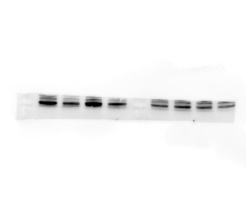


Figure2i UM-UC3 T24 SNAIL


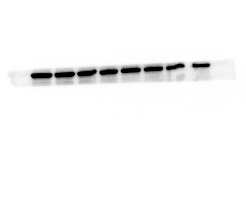


Figure2j UM-UC3 T24 actin


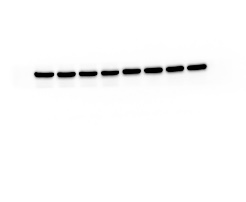


Figure2j T24 actin


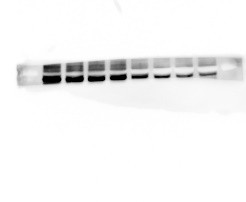


Figure2j T24 FTO


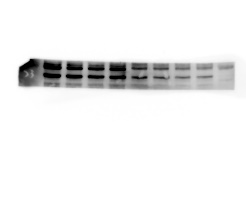


Figure2j T24 SMAD3


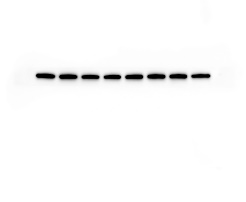


Figure2j UM-UC3 actin


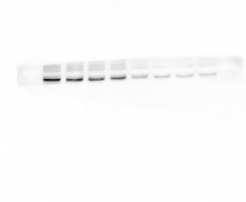


Figure2j UM-UC3 FTO


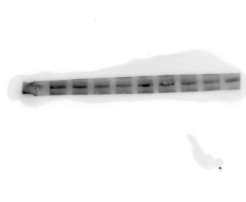


Figure2j UM-UC3 SMAD3


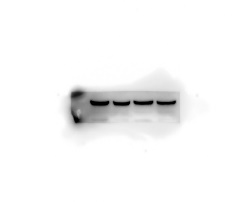


Figure3 UM-UC3 T24 actin


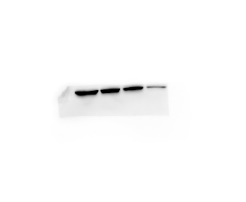


Figure3 UM-UC3 T24 CCND1


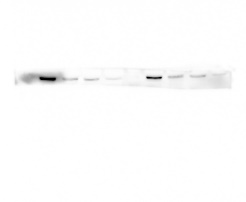


Figure3 UM-UC3 T24 FTO


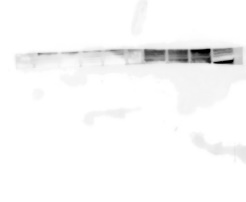


Figure3 UM-UC3 T24 MMP9


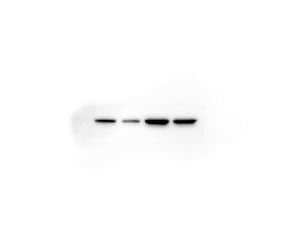


Figure3 UM-UC3 T24 CCND1 2


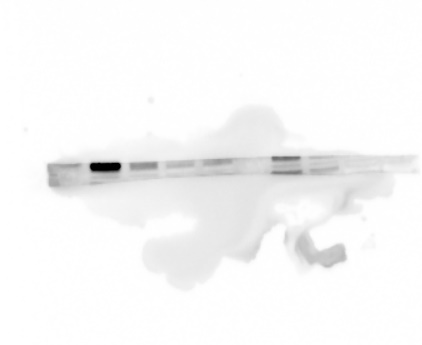


Figure3 UM-UC3 T24 MMP9 2


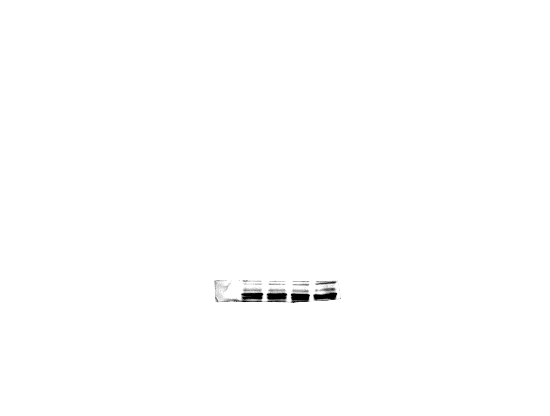


Figure 4a T24 actin


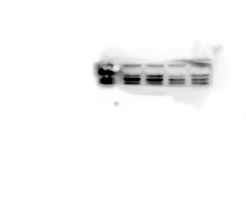


Figure 4a T24 SMAD3


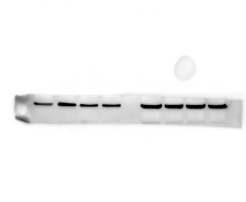


Figure4b T24 actin


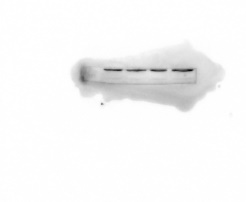


Figure 4a UM-UC3 actin


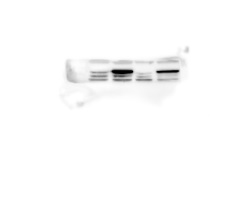


Figure 4a UM-UC3 SMAD3


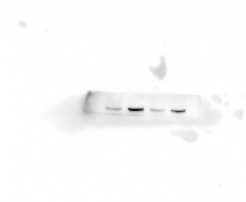


Figure 4b T24 FTO


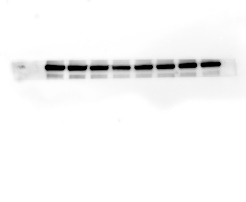


Figure4b UM-UC3 actin


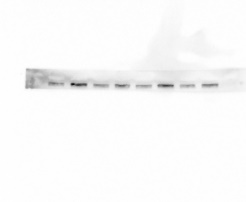


Figure4b UM-UC3 FTO


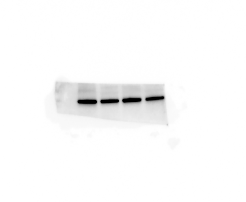


Figure5 actin


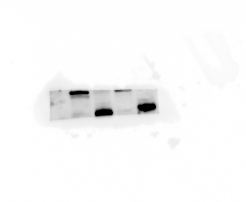


Figure5 CCND1


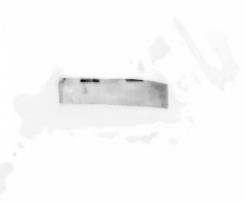


Figure 5 CDK4


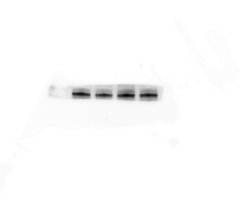


Figure 5 FTO


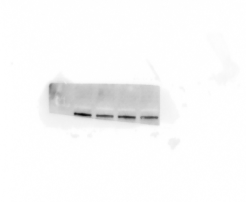


Figure 5 MMP9


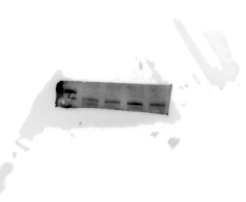


Figure 5 N-Cadherin


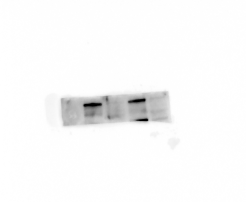


Figure 5 SMAD3


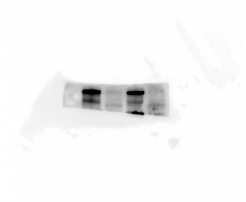


Figure 5 Vimentin
